# Measuring satisfaction with health care in young persons with inflammatory bowel disease -an instrument development and validation study

**DOI:** 10.1186/1472-6963-14-97

**Published:** 2014-03-01

**Authors:** Andrea Sadlo, Julia Altevers, Jenny Peplies, Birgit Kaltz, Martin Claßen, Alexandra Bauer, Sibylle Koletzko, Antje Timmer

**Affiliations:** 1 Division of Paediatric Gastroenterology & Hepatology, Dr. v. Hauner Kinderspital, Ludwig Maximilian University of Munich, Lindwurmstr. 4, D-80337 Munich, Germany; 2 Department of Clinical Epidemiology, Leibniz Institute for Prevention Research and Epidemiology – BIPS, Achterstr. 30, 28359 Bremen, German; 3 German Association Morbus Crohn / Colitis ulcerosa (DCCV) e.V., Bundesgeschäftsstelle, Inselstraße 1, 10179 Berlin, Germany; 4 Klinikum Links der Weser, Klinik für Kinder- u. Jugendmedizin, Senator-Weßling-Str. 1, 28277 Bremen, Germany; 5 Berufliches Schulzentrum Amberg, Abteilung Nahrung und Gesundheit, Raigeringer Str. 27, 92224 Amberg, Germany; 6 Department of Health Services Research, Division of Epidemiology and Biometry, Carl von Ossietzky University Oldenburg, D-26111 Oldenburg, Germany

**Keywords:** Patient satisfaction, Instrument development, Health care quality, Youth, Inflammatory bowel disease

## Abstract

**Background:**

Patient satisfaction is a relevant prognostic factor in young persons with chronic disease and may be both age and disease specific. To assess health care quality from the patient’s view in young persons with inflammatory bowel disease, an easy to use, valid, reliable and informative specific instrument was needed.

**Methods:**

All parts of the study were directed at persons with inflammatory bowel disease aged 15 to 24 (“youth”). A qualitative internet patient survey was used to generate items, complemented by a physician survey and literature search. A 2nd internet survey served to reduce items based on perceived importance and representativeness. Following pilot testing to assess ease of use and face validity, 150 respondents to a postal survey in patients from a paediatric clinical registry were included for validation analyses. Construct validity was assessed by relating summary scores to results from global questions on satisfaction with care using ANOVA. To assess test-retest reliability using intraclass correlation coefficients (ICC), a subset of patients were assessed twice within 3 months.

**Results:**

302 persons with IBD and 55 physicians participated in the item generating internet survey, resulting in 3,954 statements. After discarding redundancies 256 statements were presented in the 2nd internet survey. Of these, 32 items were retained. The resulting instrument assesses both the perceived relevance (importance) of an item as well as the performance of the care giver for each item for calculation of a summary satisfaction score (range 0 to 1). Sensibility testing showed good acceptance for most items. Construct validity was good, with mean scores of 0.63 (0.50 to 0.76), 0.71 (0.69 to 0.74) and 0.81 (0.79 to 0.83) for no, some and good global satisfaction (ANOVA, p < 0.001). Test-retest reliability was satisfactory (ICC 0.6 to 0.7).

**Conclusions:**

We developed an easy to use, patient oriented, valid instrument to assess satisfaction with care in young persons with IBD for use in survey research.

## Background

Quality of care in young persons with chronic disease has been receiving increasing attention over the past years [1,2]. Chronically ill adolescents and young adults are faced with the challenges of growing up and starting an independent life in the context of an often debilitating disease. At the same time, there is the additional problem of transitioning from paediatric to adult medical care which will, if poorly staged, result in a care giver gap right at the age peak in risk taking behaviour [3-6]. Consequences may be grave. As an example, non-adherence has been shown to result in a severely compromised prognosis in young transplant recipients and diabetics [7].

More disorders have by now moved into focus of care improvement programmes including gastrointestinal disease [8-10]. The inflammatory bowel diseases (IBD) comprise ulcerative colitis, Crohn’s disease, and indeterminate colitis or IBD unclassified (IBDu). These chronically relapsing diseases may occur at any age, as early as in infancy, but most commonly in adolescence and early adulthood. The bowel is the primary focus of the inflammatory process; however, any other organ may be involved. General symptoms such as weight loss, delayed growth and fatigue are common, as are issues of psychosocial maladaptation. The high frequency and variability of possible complications, the unpredictability of the course of the disease, side effects of medications as well as the often embarrassing nature of symptoms pose particularly high challenges to affected youth, their families and the attending physicians. In this situation, quality of care is essential to ensure the best possible start into adult life, both with respect to physical wellbeing as well as education, job, and family building.

Various checklists, recommendations, guidelines and consensus statements aiming to improve quality of care have been published, both general and disease specific, including several for IBD [4,9,11-14]. Still, it seems implementation lags behind considerably. In consequence, there is a lack of valid data [15-17]. This relates to the description of current health care, to patient preferences and needs, as well as to the evaluation of the effect of care improvement interventions [17].

Evaluation of quality of care is a complex issue. Classically, structural, procedural and outcome related factors should be considered [18,19]. All of these are potentially specific to the medical system, the disease at question and the age group considered. Patient satisfaction is one of several important aspects of quality of care, and possibly the one most difficult to assess and interpret due to its highly individual and subjective nature [20-22]. Patient satisfaction is a significant determinant of adherence and may as such be considered both a particularly important outcome measure as well as a relevant prognostic factor in the care of the young adult [20]. Simplistically, it may be interpreted as the assessment of structural and procedural quality from the patient’s view. There is a strong interdependency between expectations or personal values on the one hand, and perceived quality on the other hand. Also, patient satisfaction has been shown to be directly related to health status [20,23]. Thus, measuring satisfaction with care will ideally be combined with other measures of quality of care and patient wellbeing, depending on the research or policy question at hand. It should also allow for inter-individual differences in patient preferences.

A large variety of instruments is available to assess the quality of care from the patient’s view in children or adults with chronic diseases, some generic, some either age or disease specific [24-34]. Doubts remain as to the applicability and sensitivity in the specific context of IBD patients in the transitional age. For example, for the well-validated IBD specific QUOTE IBD, item generation was based on Dutch persons with a median age of 45 years [25,35]. In contrast, the widely used generic CHC-SUN, also available in German, is targeted at younger children [36]. Two methodologically particularly diligent and informative studies on patient preferences in the US and the Netherlands both focused on adolescents, but only those up to the age of 18 or 19 [6,37]. These examples illustrate a common problem in both research and patient care. By either looking primarily at children or primarily at adults youth and young adults tend to be marginalized and little is known about their specific needs and preferences.

For a comprehensive survey on the quality of care in the transitional group of IBD patients aged 15 to 24 years, we developed and tested a specific instrument to measure patient satisfaction. Patient satisfaction was conceptualized as the degree to which aspects of care which are individually perceived as important are met [20]. The instrument is meant to be used within a multi-modular questionnaire including various indicators of quality of care, sociodemographic variables and assessment of health status.

## Methods

### Study design and target population

**Figure 1:**
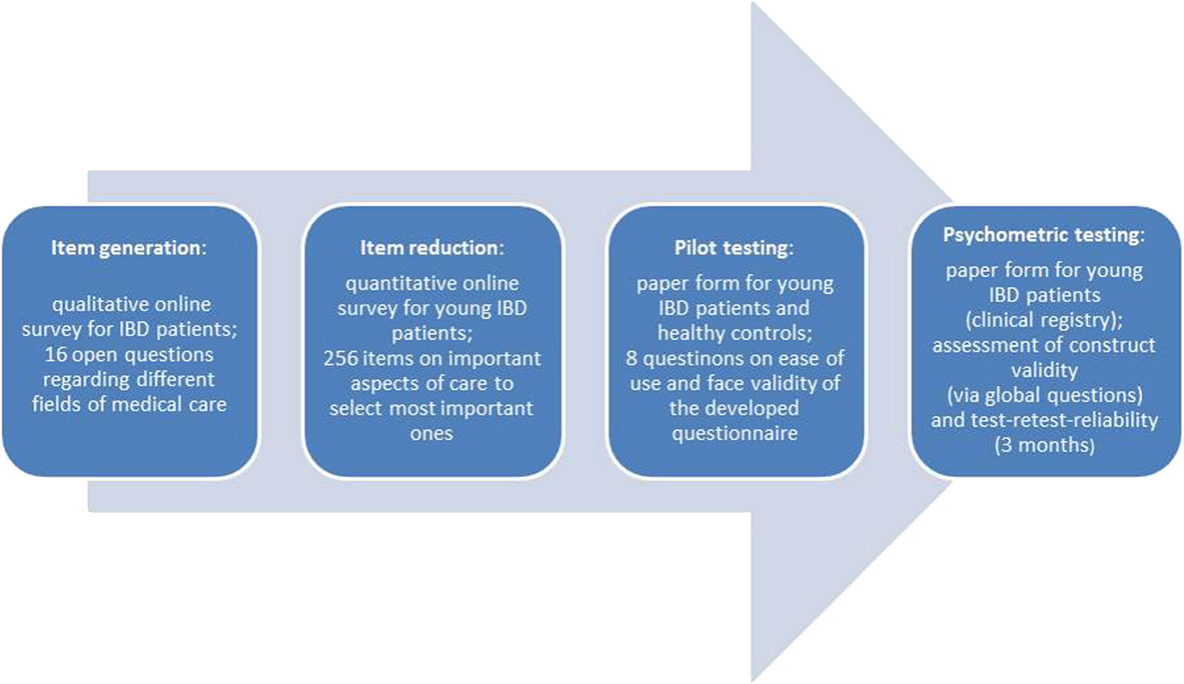
Study design – phase sequence.

We used a multistage mixed method design (Figure 1). The instrument development was based on descriptive internet surveys for item generation and reduction, followed by pilot testing using a self-employed sensibility questionnaire [38]. The validation study was based on postal questionnaires, using a cross sectional design to test construct validity and a longitudinal follow up for test-retest and sensitivity to change [39].

The target population were young persons (“youth”) with inflammatory bowel disease (Crohn’s disease, ulcerative colitis and indeterminate colitis/colitis unclassified). In accordance with the definition used by the United Nations, youth was defined as age 15 to 24 years [40].

### Questionnaire development

#### Item generation

Items were generated based on a qualitative internet survey in patients, complemented by a short qualitative physician survey and a literature search. Patients were recruited based on the youngster email lists of the national patient organizations, posters and hand-outs in doctors’ offices and outpatient departments, and internet groups focussing on IBD. Snowballing was encouraged to access persons who would otherwise not participate in survey research. All recruitment strategies served to widely distribute the internet address of an anonymous survey containing open worded questions as to what patients found important in their medical care. Questions were framed as “what do you consider important with respect to … (the treatment of your IBD, your doctor’s office, caretakers other than physicians, etc.)”.

Physicians were recruited using the email lists of the German Paediatric IBD registry, (CEDATA-GPGE, which includes paediatricians with specific interest in IBD) and the German Working Group of IBD (DACED) within the German Association of Gastroenterology (DACED, which includes researchers and gastroenterologist with specific interest in IBD, focussing on adults) and the Association of Gastroenterologists working in private practice (BNG). Overall, about 300 physicians were reached by this approach (exact numbers are not available due to overlap).

#### Qualitative analysis and item reduction

All patient statements were broken down to single items and sorted by major domains, as used by the QUOTE-IBD study group (*accessibility, costs, accommodation, continuity of care, courtesy, information, competence, patient autonomy*) [25] adding auxiliary categories as needed (*special situations in the young, therapy related issues, hospital care* and *rehabilitation*). Redundant items were removed. The resulting list was complemented by those aspects from the physician survey and literature search which were not yet covered by patient responses.

A 2nd internet survey was then performed presenting items in groups of 10 to 15 statements (1 to 4 groups of similar statements per domain). Patients were approached as before in an anonymous internet survey and were asked to select and rate from each group those statements they considered most relevant and representative. Only respondents in the target age group who had completed the full set of questions were considered in the subsequent analyses.

For the final selection, the items most often selected were retained. A more-step selection procedure ensured that items prioritized both overall and per domain/category, as well as the items most frequently selected by different age groups (15 to 17, 18 to 20 or 21 to 24 years), sex, and disease type (Crohn’s disease or ulcerative colitis/indeterminate/unclassified colitis) were included.

#### Instrument set up and pilot testing

The instrument was constructed in analogy to the QUOTE IBD and QPP questionnaires [25,31,41] as a two part questionnaire: Part A assesses the relevance of the various items as perceived by the individual respondent („importance“). Part B evaluates the performance of the current care giver as perceived by the respondent (“experience”, “reality”), both based on a 4 point answering scale.

**Figure 2:**
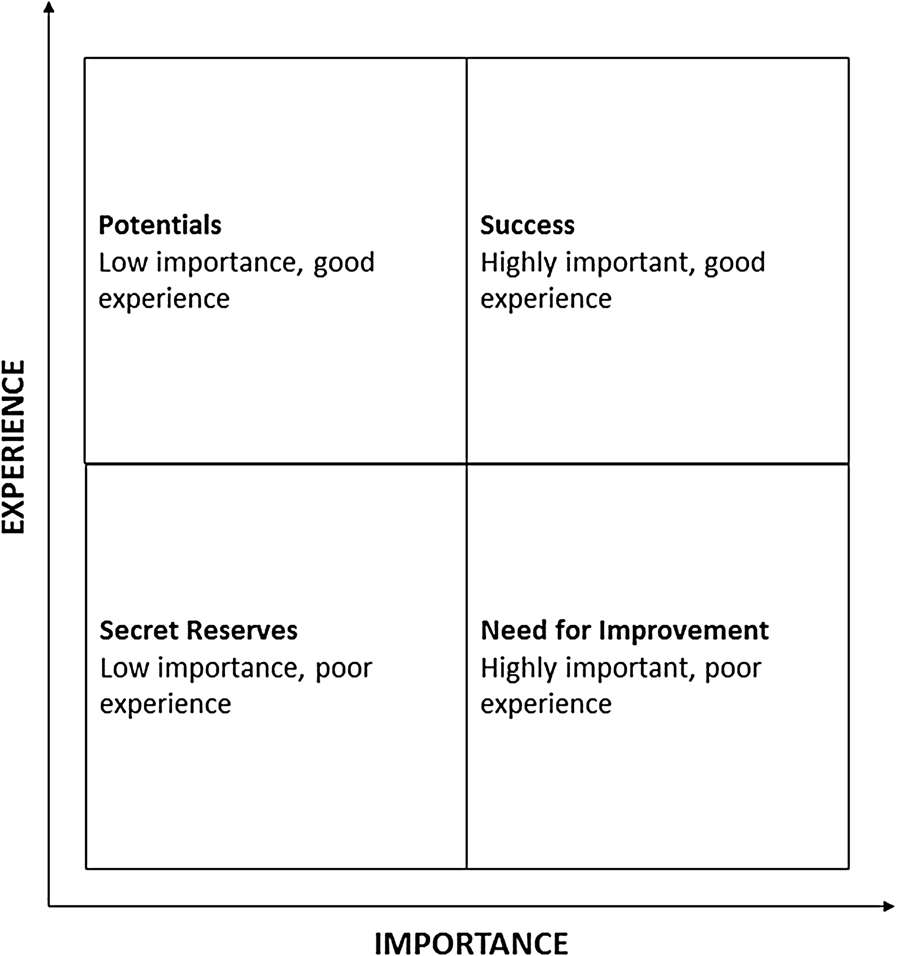
**Concept: importance - experience correlation matrix (adapted from **[42]**).**

Single item exploration may use a graphical display to correlate importance to experience, as outlined in Figure 2. A patient satisfaction summary score uses the perceived relevance (importance) of each item (part A) as a weighing factor for the corresponding item from part B. The resulting scale is linearly transformed to result in a range of 0 (completely dissatisfied) to 1 (completely satisfied). The exact algorithm is presented in the Additional file 1.

Following construction, the instrument was distributed to healthy youth (students and friends of the investigators), physicians involved in IBD care, and young patients from outpatient internist and paediatric IBD clinics. Test persons were asked to fill in a short questionnaire relating to aspects of sensibility, face and content validity such as ease of use, comprehensibility, comprehensiveness, redundancy and ability to serve the purpose at hand [38]. Answers were to be given on a 7 point scale. Mean values of 5 or more were considered sufficiently acceptable.

### Instrument validation

#### Mail survey

Validation analyses were based on the first 150 respondents of a larger survey on the situation of care in young patients with IBD performed in patients of a large paediatric clinical registry (CEDATA-GPGE) [42]. This registry was set up in 2004 by paediatric gastroenterologists and includes children and young adults with IBD from Germany and Austria. A mailed questionnaire included the patient satisfaction questionnaire and two global questions on patient satisfaction (q1: with treatment of IBD in general; q2 with the doctor primarily in charge of IBD related issues).

#### Construct validity

Summary satisfaction scores were calculated for those respondents completing both parts of the instrument for at least 24 of 32 items. For construct validity [43], scores were compared based on the results of the global questions (very satisfied, somewhat satisfied, not so/not at all satisfied), using analyses of variance.

#### Internal consistency

Internal consistency was measured by calculating Cronbach’s alpha [44], using, per item, the product of experience and importance.

#### Exploration of relevant thresholds

Various thresholds were explored e.g. based on confidence interval limits for mean values by global satisfaction. Agreement with the categories resulting from the global question on satisfaction with the IBD doctor was evaluated using Cohen’s kappa [45].

#### Test-retest reliability and sensitivity to change

For test-retest reliability, all probands from 3 major outpatient clinics who had already participated in the main survey were re-surveyed after 3 months. In addition to the satisfaction questionnaire, global questions were added relating to changes in satisfaction and disease activity status as compared to the baseline survey. The intraclass correlation coefficient (ICC) was calculated to examine agreement between base line and follow-up satisfaction summary scores [46]. A trend in mean test-retest score differences (negative for deterioration, positive for improvement), low ICC for those with changed satisfaction, and significant differences between means based on ANOVA were assumed to denote sensitivity to change.

#### General assumptions, software and quality assurance

For all statistical tests, statistical significance was assumed for p < 0.05 (two sided testing). Internet surveys were based on LimeSurvey. MAXQDA was used during the item generation phase. Sensibility testing was analysed using SPSS. The validation analyses were done in SAS 8.2 and underwent code review by a second programmer.

### Ethics

Ethical approval was sought and received from the respective institutional boards for the pilot study (LMU Munich/03-18-11) and main survey (University of Bremen/06-01-11). The internet surveys and pilot testing were anonymous, i.e. did not collect personal data nor saved IP addresses. For participation in the postal survey, written informed consent was sought from all participants and their parents if respondents were under aged. This part of the study used pseudonymized data for analysis.

## Results

### Instrument development

#### Item generation

The item generating, qualitative internet survey was open from June 22 to July 15, 2009. 302 patients participated, all of which had self-reported IBD (Table 1). 214 respondents were within the target age group (71%). Most patients were recruited via patient organization mailing (51%), followed by social network postings (22%) and physicians (20%). The physician survey was answered by 55 persons. Of these, 22 were paediatricians including 15 certified paediatric gastroenterologists, and 21 were internists, including 15 qualified gastroenterologists.

**Table 1:** Patient characteristics

	**Item generation (1st internet survey)**	**Item reduction (2nd internet survey)**	**Sensibility survey (pilot study)**	**Validation study (1st postal survey)**	**Reliability testing (2nd postal survey)**
**Source**	**Various (see text)**	**Various (see text)**	**Healthy volunteers/outpatient clinics**	**CEDATA-GPGE (clinical registry)**	**CEDATA-GPGE (clinical registry)**
Male	108 (35.8%)	24 (27.9%)	12/6	81 (54.0%)	50 (53.2%)
Female	194 (64.2%)	62 (72.1%)	18/2	69 (46.0%)	44 (46.8%)
15 to 17 years	54 (17.9%)	2 (2.3%)	8/2	61 (40.7%)	28 (29.8%)
18 to 20 years	46 (15.2%)	21 (24.4%)	8/4	77 (51.3%)	49 (52.1%)
21 to 24 years	114 (37.8%)	51 (59.3%)	13/2	11 (7.3%)	16 (17.0%)
Crohn’s disease	189 (62.6%)	55 (64%)	0/n/a	90 (60.0%)	57 (60.6%)
Ulcerative colitis	103 (34.1%)	28 (32.6%)	0/n/a	47 (31.3%)	30 (31.9%)
Indeterminate/unclassified	5 (1.7%)	3 (3.4%)	0/n/a	8 (5.3%)	3 (3.2%)
No IBD	5 (1.7%)	-	30/0	-	-
Responses overall	302	203	30/8	150	94
N in analysis	302	86	30/8	141	89

Overall, there were 3,954 statements resulting from the patient survey, which could be reduced to 225 representative items by excluding synonyms, duplicated content and overly specific observations. These patient survey derived aspects were complemented by 21 additional items from the physician survey, and 10 not yet covered aspects resulting from the literature search. The 2nd internet survey thus presented overall 256 items in 8 major and 4 auxiliary domains.

#### Item reduction

The 2nd internet survey was conducted in July and August 2010 and was filled by 203 persons. Of those completing the full set of questions (n = 115), 86 were within the target age group (75%). The items most often considered importantare presented in Table 2, including information on the source of the respective item (literature vs. expert vs. patient survey), the domain assigned by the investigator and the percentage it was chosen by participants. Patient preferences showed few differences by age and sex, and none by type of disease. Thus, only 5 items were included due to consideration of subsample priorities. One item was added as it was felt to be particularly important by the participating paediatricians (“involvement of psychologist”). Overall, the selection procedure resulted in 32 items. Of these, 29 originated from the patient survey, 2 from the physician survey, and 1 from an internet based doctor’s checklist (Table 2). Three items were clearly diseasespecific, relating to the availability of toilets, to colonoscopy preparation and to treatment with corticosteroids. All other items were either clearly generic, with a particular weight on physician-patient interaction, or related to generic issues phrased in a disease specific way. The complete questionnaire is available on request in German. An ad hoc translated English version is included as an Additional file 2.

**Table 2:** Selected items for the final questionnaire, based on frequency of selection by the participants (n = 86)

**Item**	**Origin**	**Category**	**Content**	**Frequency of selection in %**	**Reason for selection**
1	P	A	Appointments compatible with school/working schedule	33.7	
2	P	A	Same day emergency appointment	72.1	Top 2
3	P	CO	No difference based on insurance status	65.1	Top 3
4	P	CO	Easy access to follow-up prescriptions	45.3	
5	P	CO	No unnecessary investigations	41.9	
6	P	CO	Liquid laxative with acceptable taste	46.5	
7	P	CO	Share experiences with other patients	43.0/51.2	
8	P	CO	Treatment in specialized hospitals	51.2	
9	P	AC	Clean and hygienic office	73.3	Top 1
10	P	AC	Sufficient number of clean restrooms	43.0	
11	P	CC	Treatment always by the same doctor	48.8	
12	D	CC	Cooperation of doctor with IBD clinics	44.2	
13	D	CC	Smooth communication between GP and IBD doctor	41.9	
14	P	CC	Involvement of psychologist/psychotherapist	65.1	Expert opinion
15	P	CC	Involvement of other specialized doctors	76.7	
16	P	C	Individual treatment	53.5	
17	P	C	Understanding of fears and worries	54.7	Female
18	P	C	Discusses investigations/results	50.0	
19	P	C	Taking personal situation into account	47.7	Age 18-20
20	P	C	Devoting enough time	39.5	Age 15-17
21	P	C	Showing that it is important that the patient gets better	44.2	
22	P	C	Bearing personal life planning in mind	38.4/39.5/32.6	
23	P	C	Friendly and polite nurses	57.0	
24	P	C	Understanding and respectful nurses	55.8/53.5	
25	P	I	Explanations in easy-to-understand language	25.6	Age 15-17
26	P	I	Information about investigations/results	43.0/58.1	
27	P	I	Listening/responding to problems	46.5	
28	P	CM	Experience in IBD treatment	52.3	
29	P	CM	up to date knowledge about IBD	45.3	
30	P	CM	Alternatives to steroid treatment	40.7	Age 18-20
31	P	AU	Coordination of therapeutic decisions	48.8	
32	L	AU	Facilitates 2nd opinion	46.5	

Explanation: If two or three items were collapsed into one, the frequencies of all original items are shown (see Item 7, 22, 24, and 26). If an item was selected because of top preference in a subgroup (sex, age), this is documented in the last column. P = Qualitative Patient Survey, D = Qualitative Doctor Survey, L = Literature. A = Accessibility, CO = Costs and Organization, AC = Accommodation, CC = Continuity of Care, C = Courtesy, I = Information, CM = Competence, AU = Autonomy.

#### Pilot testing

30 healthy persons and 8 patients with IBD handed in a formally completed sensibility questionnaire. Consulting physicians gave informal feedback. The time to complete the instrument ranged from 5 to 25 min (mean 12.2 min). Acceptance was problematic with respect to the answer categories offered (mean 4.5) and to comprehensiveness (4.9). The wording of the answer categories was subsequently modified. All other aspects were considered acceptable by all groups. Ease of use was considered particularly excellent (mean 6.5). All ratings below 5 came from IBD patients. They were generally slightly more critical than healthy volunteers.

### Validation survey

#### Descriptive analyses: single item exploration and summary satisfaction score

141 persons answered the questions on perceived importance. Most items were considered important or extremely important by the majority of respondents. These categories were chosen on average by 55 (39%) and 67 (48%) of cases, respectively. Similarly, experience was mostly rated as generally or fully meeting expectations, although for this part, the distribution was slightly more balanced (met 47 (33%), fully met 66 (47%)).

In analogy to the distribution for single items, summary scores were also skewed towards favourable results. Results per case ranged from 0.28 to 0.98, both overall median and mean were 0.76 (SD 0.12), median interquartile range was 0.68 to 0.84.

#### Construct validity

**Figure 3:**
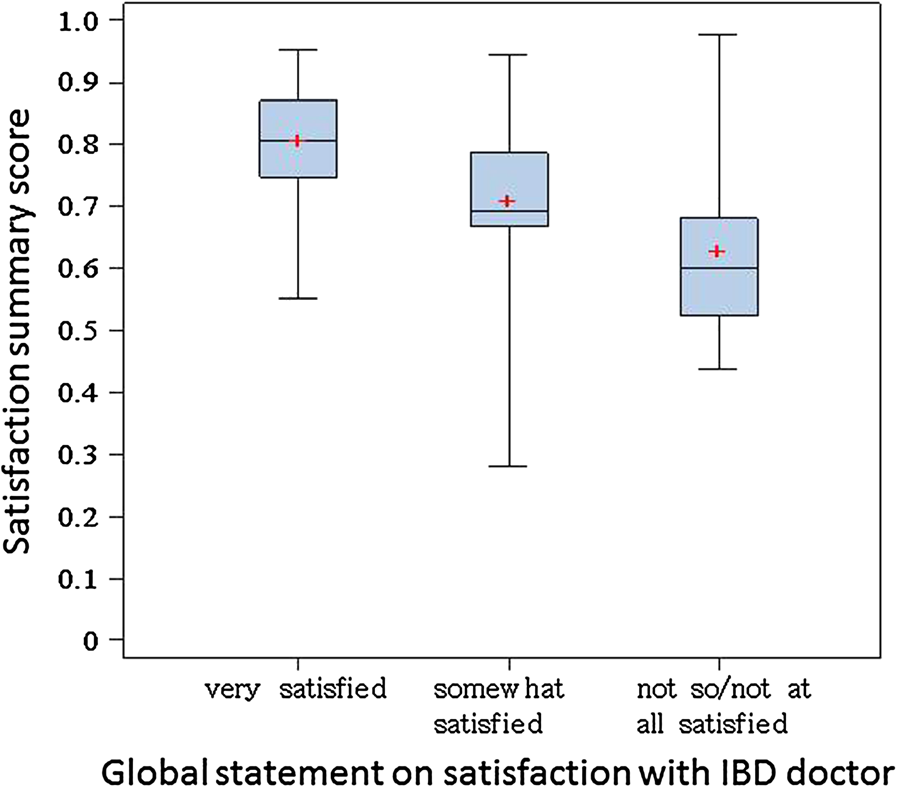
Satisfaction scores – by global satisfaction category.

Global questions were answered by 138 (q1) and 135 (q2) persons respectively. To both questions, only 2 persons each answered “very unsatisfied”, and this category was therefore combined with the “somewhat dissatisfied” category. This resulted in comparison group sizes of 13-64-61 persons (q1; lowest to highest) and 9-56-70 (q2), respectively. The distribution of scores (boxplots) is shown in Figure 3. The respective mean satisfaction scores were 0.67 (0.58 to 0.77), 0.73 (0.70 to 0.75) and 0.81 (0.78 to 0.83) for q1 (ANOVA, p < 0.001), and 0.63 (0.50 to 0.76), 0.71 (0.69 to 0.74) and 0.81 (0.79 to 0.83) for q2 (ANOVA, p < 0.001).

#### Internal consistency

Cronbach’s alpha based on 141 respondents was calculated as 0.87, indicating high average item inter-correlation (good internal consistency).

#### Evaluation of useful thresholds

Tertiles were calculated to be < 0.70, 0. 70 to 0.81 and > 0.81. Both the middle and the high tertile are well within the range of high satisfaction. Fair agreement with the global question on satisfaction with the IBD doctor was found using the thresholds > 0.7 (very satisfied), 0.55 to 0.7 (satisfied) and < 0.55 (not satisfied) (Kappa 0.5).

#### Test-retest reliability and sensitivity to change

**Figure 4:**
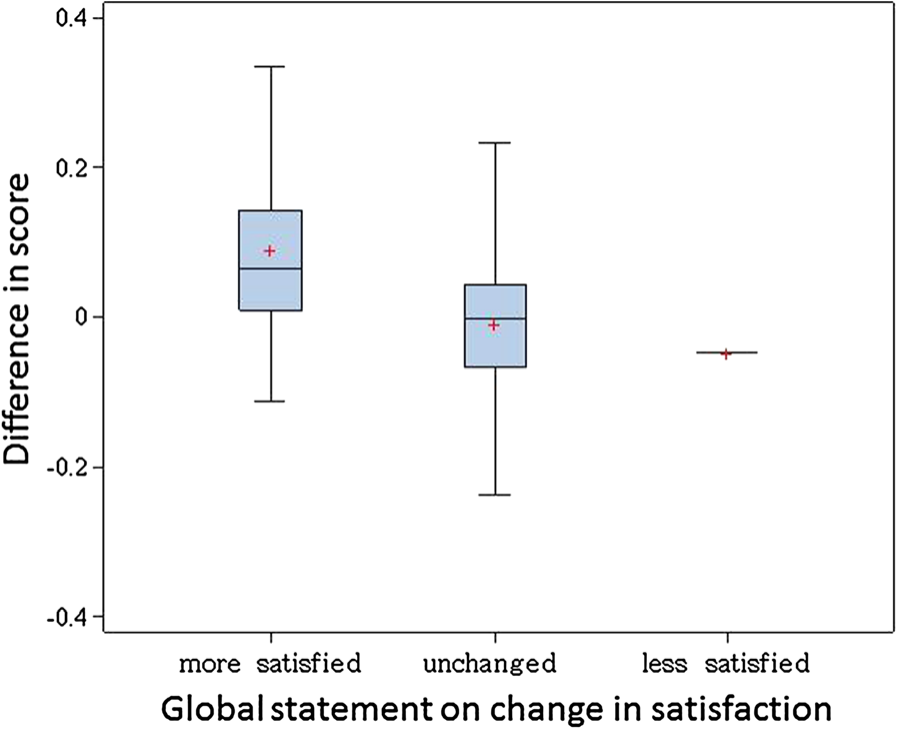
Distribution of mean differences (test-retest scores) – by change in satisfaction o.

94 of 141 (65%) persons sent back the follow up questionnaire, including 89 with sufficient item response for reliability analysis. Of these, 74 (83%) reported unchanged satisfaction with IBD care, 13 were better, 1 was worse. The boxplots of satisfaction scores by change in satisfaction is shown in Figure 4. Mean differences were - 0.01 (95% CI 0.01 to −0.03) for those unchanged and 0.09 (95% CI 0.01 to 0.17) for those who perceived better care (single person with worse care: − 0.05) (ANOVA, p = 0.01). There was no statistical difference in changes to the satisfaction care by change in disease activity (− 0.01 vs. -0.02 vs. – 0.04, p = 0.43). The ICC was 0.6 for those with unchanged satisfaction, and 0.7 if disease activity was also stable. Those with better or worse satisfaction had an ICC of 0.5, irrespective of changes in disease activity.

## Discussion

We developed an easy to use instrument with good construct validity and satisfactory test-retest reliability to assess patient satisfaction with quality of care in young persons with IBD. Reviewers have previously highlighted both the high inter-individual variation as well as the “dynamic two-way nature” of patient satisfaction as the “difference between expectations and perceptions” [20]. This concept is mirrored by our two step approach, as we used the importance patients attach to certain aspects of quality of care to weigh how they rated their experience of care. The structural analogy to similar instruments established in the assessment of adult IBD populations is expected to facilitate acceptance by patient representatives and professionals actively engaged in the improvement of care in these diseases [25,35,47].

The pilot study (sensibility survey) showed good acceptance of the instrument.

In accordance with findings from other studies on patient perceived quality of care [26], physician competence and empathy were found to be particularly important and are represented accordingly in the instrument. There is some indication that patient satisfaction has marked disease and, possibly, system specific aspects. For example, other than reported from the US or Great Britain [48] it was evident that waiting times and costs do not play a major role in young patient concerns in Germany and Austria. This is in accordance with informal information from providers in Germany and Austria, and has also been reported from the Netherlands [4].

A major strength of this study is the rigorous inclusion of the young chronic patient perspective. Patient participation in the development of instruments on patient related outcomes is now generally recommended [20,49]. To get an as wide as possible spectrum of different patients, we used a very broad access during the item generating and reduction phase, including multiple recruitment strategies. In particular, by using approaches via the internet and keeping the survey completely anonymous we expected to be able to include youth who might otherwise not have participated. The number and variability of responses by far exceeded our expectation. Also, we were impressed by the very constructive and helpful attitude shown by all respondents, resulting in a wealth of different statements.

We placed a special focus on the quality of the disease specific physician care, reflected by a higher agreement with the global question on satisfaction with the physician in care of the IBD, as compared to the global question on IBD treatment in general. This is in contrast to other instruments, such as the generic Child Health Care Questionnaire on Satisfaction, Utilization and Needs (CHC-SUN) targeted at younger children, which applies a more general approach to medical care [36].

The instrument facilitates to pinpoint specific areas for improvement based on single item description which may be given as a feed back to the care giver. For example, in our on-going study on the quality of care in the transitional stage, all outpatient departments contributing 20 or more patients will receive the item based correlation matrix as well as benchmarked single item performance. Of note, validation has only been performed for the summary score. In consequence, single item based presentations should be reserved for individual descriptive purposes as suggested.

### Limitations

Satisfaction with care is a difficult concept, and some have doubted whether it can be validly assessed at all [20]. We appreciate that the approach we chose is just one way of tackling this issue. Results may not be used as a direct measure of quality of care, but should be viewed as one of several aspects. Combination with complementary measures of care including those of structural and procedural quality, as well as assessment of health status is recommended to aid interpretation.

Our instrument underwent detailed tests for validation in independent data sets, following well established recommendations [39,43,49]. In contrast, the procedures we used for instrument development deviate in several aspects from standard methods commonly reported. For example, items were generated from open, qualitative internet surveys rather than resorting to, for example, personal expert interviews or patient focus groups [50]. A broad approach as outlined above was necessary in order to reach a sufficiently diverse patient group which would be truly independent from the subsequent test sample and study population. Unfortunately, so far, there is little empirical evidence on the pros and cons of internet surveys in this context [20]. Similarly, we used content oriented quota sampling rather than data driven methods to reduce the items. This was, besides being owed to personal preference [51], in part a consequence of the amount of statements received and the format in which they were available. For example, assumptions were not met to perform factorial analysis. We wish to stress however, that so far there is no evidence that any specific method would have been superior [52], nor are there standard rules on how item reduction should be performed [20,49]. As there was no formal derivation or testing of specific domains, the use of sub-scores is discouraged. Overall, the test performance as reported above proved our approach to be successful, with some minor restrictions:

Comprehensiveness of the instrument was critically assessed by some IBD test persons during the pilot study. We are afraid that this is inherent to the topic, as shown by the large amount of different issues raised by the respondents to the 1st internet survey. Any selection of items will have to be a compromise. Our instrument allows for the appreciation of inter-individual variation by employing weights based on perceived importance of the different items, as used by similar measures [25,41]. Still, we suggest, that any study on the satisfaction with care should encourage additional free text comments to accommodate the individual nature of patient preferences.

Another problem relates to a marked ceiling effect. Some of this may be due to our mode of sampling which is likely to have aggravated responder bias. In order to achieve high and timely cooperation rates, we used the first respondents of a larger survey for this validation study. It has been shown that in general, both the particularly satisfied as well as the particularly dissatisfied tend to be more responsive in studies on patient satisfaction [20]. However, in our case we may have oversampled persons with high satisfaction. This was also observed for other instruments, such as the child ZAP [30]. Of note, for the QUOTE IBD, a 90% satisfaction rate was assumed normal [25,35]. We will rerun the analyses on the distribution of items when using the instrument in other contexts.

While the representativeness of the results is usually a minor concern in studies on instrument development and validation [39], the low numbers of less satisfied patients posed specific problems with insufficient power when examining agreement of categorized variables and sensitivity to change. There is some indication that stability of patient satisfaction is relatively high and may be more influenced by disease activity rather than actual changes, but more information on this is certainly warranted.

### Other practical implications

Our instrument was specifically designed to assess patient satisfaction with IBD physician care. Patient satisfaction, however, is only one element, or outcome, of successful transition, and the physician is only one player determining good quality care. Evolvement of knowledge, self-management skills and maturation in attitudes and behaviour are all necessary to enable the patient to successfully master the challenges of adult life with chronic disease. Owing to the high importance currently attached to transitional care, instruments and surveys are increasingly available for these many different purposes, including the description of the transition process as such [29,48,52-54]. We acknowledge that for some aspects qualitative approaches may be particularly helpful [20,48]. Naturally, the choice of the instrument will depend on the specific study question at hand.

## Conclusion

This newly developed instrument is expected to be a useful tool in the assessment of quality of care from the patient’s perspective in young persons with IBD. It is currently used in two on-going studies on patient care in the transitional stage. More information will be collected on the performance of the instrument in these more heterogeneous patient samples.

### Note

This manuscript is based in part on the theses of Andrea Sadlo (Diploma in Psychology, LMU Munich) and Julia Altevers (BA Public Health, University of Bremen). Preliminary results were presented at Annual Meetings of the German Epidemiological Association (DGEpi) (2011, instrument development, 2012, validation) and various working group sessions (DACED 2011, CEDATA-GPGE 2012).

## Competing interests

The authors declare that they have no competing interests.

## Authors’ contributions

AS planned, set up and analysed the 2nd internet survey, performed the item reduction, set up the final instrument, performed and analysed the pilot study, drafted the manuscript and worked on the reviewer suggestions. JA planned and performed the validation analyses and drafted the manuscript. She also participated in data editing and data entry. JP coordinated the validation survey, data entry and editing. BK consulted on the planning of all surveys, and the interpretation, and facilitated patient access. AB analysed the 1st internet survey and participated in the item reduction. MC coordinated the input of the German Language Paediatric IBD registry (CEDATA GPGE), consulted on the paediatric content and recruited patients during all stages of the project. SK served as the paediatric gastroenterology expert, participated in the expert survey and recruited patients during all stages of the project. AT conceived and coordinated all stages of the project, supervised the theses, and wrote and revised the manuscript. All authors read and contributed to the manuscript and approved of the final version.

## Pre-publication history

The pre-publication history for this paper can be accessed here:

http://www.biomedcentral.com/1472-6963/14/97/prepub

## Supplementary Material

Additional file 1 Algorithm for calculating summary score. Click here for file

Additional file 2 **Satisfaction questionnaire (English version, ad hoc translation).** Click here for file
