# Supplementary material for: Measuring satisfaction with health care in young persons with inflammatory bowel disease -an instrument development and validation study
Source: BMC Health Serv Res. 2014 Mar 1;14:97. doi: 10.1186/1472-6963-14-97 (PMC3946022; doi:10.1186/1472-6963-14-97)
Supplement: Additional file 1 — Algorithm for calculating summary score. [file 1472-6963-14-97-S1.docx]

**Calculation of the patient satisfaction summary score**

In order to calculate a patient satisfaction summary score, the following values are assigned to these 4 point scales:

- Part A (“importance”, “relevance”)
  - not important = 1
  - less important = 2
  - important = 3
  - extremely important = 4
- Part B (“experience”, “reality”)
  - not meeting expectations = 1
  - somewhat meeting expectations = 2
  - generally meeting expectations = 3
  - fully meeting expectations = 4

A crude summary score is computed using the following formula:

$patient satisfaction summary score \left( crude \right)= \frac{\sum_{i=1}^{32} \left( importance * experience \right)}{\sum_{i=1}^{32} \left( importance * 4 \right)} where i=item$

To obtain a range of 0 (completely dissatisfied) to 100 (completely satisfied), this scale is then linearly transformed:

$patient satisfaction summary score= \frac{(crude summary score - 0.25)}{0.75}$
