# Supplementary material for: Measuring satisfaction with health care in young persons with inflammatory bowel disease -an instrument development and validation study
Source: BMC Health Serv Res. 2014 Mar 1;14:97. doi: 10.1186/1472-6963-14-97 (PMC3946022; doi:10.1186/1472-6963-14-97)
Supplement: Additional file 2 — Satisfaction questionnaire (English version, ad hoc translation). [file 1472-6963-14-97-S2.pdf]

Satisfaction with your medical care.

Part A: What do you expect from your medical care?
We would like to know what matters to you, personally, about your medical care.
We will present you with a number of statements. Please tell us how important each statement is for you by ticking the box that you think fits best.

		Not important	Less important	Important	Very important
		1	2	3	4
1	My IBD doctor should take into account my school / job time tables when scheduling appointments if possible.	O	O	O	O
2	In emergency situations, I should get an appointment on the same day.	O	O	O	O
3	My IBD doctor should not make a difference based on type of insurance (private vs. statutory), e.g. regarding waiting times or medical examinations offered.	O	O	O	O
4	Follow-up prescriptions should be easy to attain (e.g. prescribed by my GP, via mail or in cooperation with my local pharmacy).	O	O	O	O
5	There shouldn't be unnecessary examinations (e.g. no colonoscopy unless it's absolutely essential).	O	O	O	O
6	The liquid laxative for colonoscopies should taste tolerable.	O	O	O	O
7	I should to be able to exchange experiences with people my age and other IBD patients (e.g. in hospital or at health resorts.)	O	O	O	O
8	I should be treated in hospitals (respectively health resorts) specialised in IBD.	O	O	O	O
9	My IBD doctor should work in clean and hygienic premises.	O	O	O	O
10	The doctor's office/outpatient clinic should have a sufficient number of clean restrooms.	O	O	O	O
11	I should be always treated by one and the same doctor at the doctor's office/outpatient clinic.	O	O	O	O
12	My IBD doctor should be cooperating with experienced IBD clinics, respectively with a university hospital.	O	O	O	O
13	The communication between my GP and my IBD doctor should flow smoothly.	O	O	O	O
14	My IBD doctor should have a psychologist / psychotherapist involved in my treatment.	O	O	O	O

		Not important	Less important	Important	Very important
		1	2	3	4
15	Other specialists should be involved in my treatment (dermatologists, ophthalmologists, rheumatologists).	O	O	O	O
16	My IBD doctor should be listening and responding to me individually.	O	O	O	O
17	My IBD doctor should understand the fears and worries that come with this kind of disease.	O	O	O	O
18	My IBD doctor should talk to me openly about problems and diagnoses.	O	O	O	O
19	My IBD doctor should take into account my current personal and job situation to find the best solution.	O	O	O	O
20	My IBD doctor should devote enough time.	O	O	O	O
21	My IBD doctor should inspire the feeling that it is important to him/her that I feel better.	O	O	O	O
22	My IBD doctor should bear my personal plans for life in mind (education/training, plans for family and future).	O	O	O	O
23	The nurses at the doctor’s office/ outpatient clinic should be friendly and polite.	O	O	O	O
24	The nurses at the doctor’s office/ outpatient clinic should treat me and my illness with understanding and respect.	O	O	O	O
25	My IBD doctor should explain the disease, treatment etc. in a way which is easy to understand also for young persons.	O	O	O	O
26	My IBD doctor should inform me about upcoming examinations and results.	O	O	O	O
27	My IBD doctor should listen and respond to my problems at length.	O	O	O	O
28	My IBD doctor should have sufficient experience in treating IBD patients.	O	O	O	O
29	My IBD doctor’s knowledge about IBD should be up to date.	O	O	O	O
30	My IBD doctor should not generally resort to steroids but consider other options.	O	O	O	O
31	I should be involved in decision making with respect to my treatment.	O	O	O	O
32	My IBD doctor should accept it if I wish to get a second opinion.	O	O	O	O

Part B. What is your experience?

Part A was about learning what matters to you about your medical care.
Now, in Part B, we would like to learn about your personal experience in medical care, once more using the statements we established in Part A. So this part is about evaluating your IBD doctor/ the doctor currently taking care of your IBD treatment.

		No	Not really	In the whole, yes	Yes
		1	2	3	4
1	My IBD doctor takes into account my school / job timetables when scheduling appointments if possible.	O	O	O	O
2	In emergency situations, I get an appointment on the same day.	O	O	O	O
3	My IBD doctor does not make a difference based on type of insurance (private vs. statutory), e.g. regarding waiting times or offered medical examinations.	O	O	O	O
4	Follow-up prescriptions are easy to attain (e.g. prescribed by my GP, via mail or in cooperation with my local pharmacy).	O	O	O	O
5	There are no unnecessary examinations (e.g. no colonoscopy unless it's absolutely necessary).	O	O	O	O
6	The liquid laxative for colonoscopies tastes tolerable.	O	O	O	O
7	I am able to exchange experiences with people my age and other IBD patients (e.g. in hospital or at health resorts).	O	O	O	O
8	I am treated in hospitals (respectively health resorts) specializing in IBD.	O	O	O	O
9	My IBD doctor works in clean and hygienic premises.	O	O	O	O
10	The doctor's office / outpatient clinic has a sufficient number of clean restrooms.	O	O	O	O
11	I am always treated by one and the same doctor at the doctor's office / outpatient clinic.	O	O	O	O
12	My IBD doctor is cooperating with experienced IBD clinics, respectively with a university hospital.	O	O	O	O
13	The communication between my GP and my IBD doctor flows smootly.	O	O	O	O
14	My IBD doctor has a psychologist / psychotherapist included in my treatment.	O	O	O	O

		No	Not really	In the whole, yes	Yes
		1	2	3	4
15	Other specialists are involved in my treatment (dermatologists, ophtalmologists, rheumatologists).	O	O	O	O
16	My IBD doctor is listening and responding to me individually.	O	O	O	O
17	My IBD doctor understands the fears and worries that come with this kind of disease.	O	O	O	O
18	My IBD doctor talks to me openly about problems and diagnoses.	O	O	O	O
19	My IBD doctor takes into account my current personal and job situation to find the best solution.	O	O	O	O
20	My IBD doctor devotes enough time.	O	O	O	O
21	My IBD doctor inspires the feeling that it is important to him / her that I feel better.	O	O	O	O
22	My IBD doctor bears my personal plans for life in mind (education / training, plans for family and future).	O	O	O	O
23	The nurses at the doctor’s office / outpatient clinic are friendly and polite.	O	O	O	O
24	The nurses at the doctor’s office / outpatient clinic treat me and my illness with understanding and respect.	O	O	O	O
25	My IBD doctor explains the disease, treatment etc. in a way which is easy to understand also for young persons.	O	O	O	O
26	My IBD doctor informs me about upcoming examinations and results.	O	O	O	O
27	My IBD doctor listens and responds to my problems at length.	O	O	O	O
28	My IBD doctor has sufficient experience in treating IBD patients.	O	O	O	O
29	My IBD doctor’s knowledge about IBD is up to date.	O	O	O	O
30	My IBD doctor does not generally resort to steroids but considers other options.	O	O	O	O
31	I am involved in decision making with respect to my treatment.	O	O	O	O
32	My IBD doctor accepts if I wish to get a second opinion.	O	O	O	O
